# Protective Effects of Pectin From Honey‐Processed Hawthorn on Acute Myocardial Ischemia

**DOI:** 10.1002/fsn3.70764

**Published:** 2025-08-11

**Authors:** Jing Li, Meiqi Liu, Yu Zheng, Qian Cai, Baojie Zhang, Yang Qu

**Affiliations:** ^1^ College of Pharmacy Liaoning University of Traditional Chinese Medicine Shenyang Liaoning China

**Keywords:** acute myocardial ischemia, gut microbiota, honey‐processed hawthorn pectin, processing of hawthorn, short‐chain fatty acid

## Abstract

Previous studies have shown that both hawthorn and honey‐processed hawthorn (HH) can prevent heart injury in acute myocardial ischemia (AMI). Hawthorn pectin (HP) and stir‐fried hawthorn pectin (FHP) have been identified as protective agents against AMI, with the effects closely related to their structural characteristics. This study aims to investigate the effects of pectin of HH (HHP) in preventing AMI. Results showed that the esterification degree, the content of galacturonic acid, and the viscosity‐average molecular weight of HHP were lower than those of HP. Rats pretreated with HHP exhibited improvements when suffering from AMI in maintaining the levels of myocardial enzyme as well as indicators related to oxidative stress. In terms of gut microbiota and short‐chain fatty acids, HHP enriched Monoglobus and increased the content of propionic acid, while HP enriched Lachnospira and increased the content of acetic acid in the intestine. The different effects of HHP from HP on regulating gut microbiota and short‐chain fatty acids might be attributed to structural alterations of the HHP after processing of hawthorn. These results contributed to the established connection between pectin consumption and its protective effect from AMI.

## Graphical abstract

Pectin prophylaxis improved myocardial enzyme levels and oxidative stress indicators in AMI rats, with hawthorn pectin enriched Lachnospira and increased acetic acid content, while honey‐processed hawthorn pectin enriched Monoglobus and increased propionic acid content.

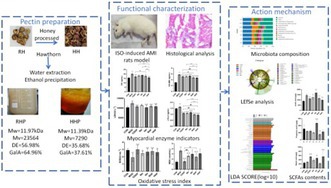

## 1 Introduction

Cardiovascular disease (CVDs) is the leading cause of death globally, with a resurgence in high‐income countries and an ongoing rapid rise in low‐and middle‐income countries (Roth et al. 2020; Timmis et al. 2020; Virani et al. 2020). CVDs are defined as diseases associated with abnormal heart and blood vessel functions, such as hypertension, atherosclerosis (AS), heart failure (HF), coronary heart disease, and coronary artery disease (Daiber et al. 2019; Figtree et al. 2021). Acute myocardial ischaemia (AMI), as a representative pathological process of CVDs, involves multiple mechanisms including energy deficit, inflammation reaction, oxidative stress, and so on (Yang et al. 2022; Zheng et al. 2022). Most CVD risk factors induce dysbiosis, which is associated with intestinal inflammation and reduced gut barrier integrity. The elevated levels of gut bacteria‐derived structural and microbial metabolites in the circulation thereby accelerate CVD development (Battson et al. 2018). This additional connection between the gut and the heart, that is, the “gut‐heart axis” (Zhao et al. 2023).

Hawthorn belonging to the genus *Crataegus* of the Rosaceae family is consumed as fruit and is used in many desserts and drinks, such as teas, candy, canned fruit, and food supplements (Li et al. 2022; Yang and Liu 2012). Hawthorn has been used as a traditional medicine in China for a long time. Its use in treating cardiovascular diseases (CVDs) began at the end of the 19th century (Wu et al. 2020). Previous studies showed that the bioactive components of hawthorn that effectively acted on CVDs mainly included flavonoids, polyphenols, and oligomeric proanthocyanidins, which could inhibit inflammation and oxidative stress (Han et al. 2016). Our previous research revealed that pectin from hawthorn (HP), with the content of 5% ~ 29% (Zhou et al. 2021), played a protective role in acute myocardial ischemia (AMI) by regulating intestinal flora and short‐chain fatty acids (Lou et al. 2024). After stir‐frying, the pectin with a high esterification degree (DE) in hawthorn transformed to low DE pectin, with a lower content of galacturonic acid (GalA) and viscosity‐average molecular weight (Zhang et al. 2023). The effects of pectin from stir‐fried hawthorn (FHP) on the indexes of creatine kinase (CK), creatine kinase MB (CK‐MB) and cardiac troponin I (cTn‐I) of AMI rats showed a better tendency than those of HP, indicating the structural changes of pectin during the processing of hawthorn led to the enhancement of its ischemic protective effect (Lou et al. 2024).

Honey‐processing is a traditional Chinese medicine processing method, which could change the composition and efficacy of medicine. Our previous research revealed that honey‐processing changed the composition of organic acids, phenylpropanoids, and flavonoids in hawthorn. The regulation on myocardial enzyme as well as indicators related to oxidative stress on AMI rats of the decoction of honey‐processing hawthorn (HH) was better than that of hawthorn (Ao et al. 2020).

Since preliminary research on the cardioprotective effects of HH mainly focused on compounds with low molecular weight the structural changes of pectin might also occur in the honey processing of hawthorn. Therefore, this study aims to investigate the structure of HHP and its cardioprotective effects, attempting to clarify the relationship between pectin structure and these effects.

## 2 Materials and Methods

### 2.1 Materials

The raw hawthorn slices (RH, batch no. 1220630) were purchased from Shaohuatang Sinopharm co. Ltd. (Bozhou, Anhui, China). Honey‐processed hawthorn (HH) was prepared based on the literature (Ao, Qu, et al. 2020).

The contents of total flavonoid (TFC) of RH and HH were determined by the colorimetry method using sodium nitrite‐aluminum nitrate as chromogenic agent (De Souza and De Giovani 2005). The contents of organic acid (OAC) of them were determined according to the titration method described in the Chinese Pharmacopeia (“Pharmacopoeia of People's Republic of China,” 2020). The contents of reducing saccharides (RSC) of them were determined by the 3, 5‐dinitrosalicylic acid (DNS) method (Krivorotova and Sereikaite 2014). While the contents of total phenol (TPC) were determined according to the Folin–Ciocalteu method (Dominguez‐López et al. 2024).

### 2.2 Extraction and Structural Characterization of Pectin

Powder of RH and HH was mixed with water at a ratio of 1:20 and refluxed at 90°C for 3 h. Subsequently, the resulting supernatant was precipitated using a 95% ethanol (at a ratio of 1:2 v/v) for 24 h at 4°C. The precipitate was washed with anhydrous ethanol twice. Finally, the precipitate was vacuum‐dried at 60°C and freeze‐dried to yield pectin of RH and HH, that is, RHP and HHP, respectively.

The molecular weight (Mw) of RHP and HHP was determined by High Performance Gel Permeation Chromatography (HPGPC) on an Agilent 1260 Series HPLC System with TSKgel G4000PWXL gel column (7.8 × 300 mm). The mobile phase was 150 mM PBS buffer with a flow rate of 0.5 mL/min; the temperature was 35°C. The standard curve was drawn by glucan kit with different molecular weights.

The DE of RHP and HHP was determined by the titrimetric method (Raji et al. 2017). In addition, the meta‐hydroxydiphenyl approach was employed to measure the content of GalA (Blumenkrantz and Asboe‐Hansen 1973). The viscosity‐average molecular weight (Mv) of RHP and HHP was determined by a Ubbelohde viscometer (Xu et al. 2023).

### 2.3 Animal Model and Drug Administration

All the procedures were approved by the Animal Care and Use Committee of the Liaoning Institute of Materia Medica (Liaoning, China). Sprague–Dawley male rats (certificate No. SYXK (Liaoning) 2020‐0001, Liaoning, China), weighing 200 ± 20 g, were purchased from Changsheng Biotechnology Co. Ltd., Liaoning, China. The animals were housed in an air‐conditioned room at 22°C ± 2°C. Standard animal chow and water were freely available. After 1 week of adaptation, the rats were randomly assigned to control (CON), model (MOD), positive control (Compound Danshen Dripping Pills, CDDP), RH, HH, RHP, and HHP groups (*n* = 6). The CDDP group was administered at a dose of 85 mg kg^−1^, the RH and HH groups were administered RH and HH at a dose of 1260 mg kg^−1^, the RHP and HHP groups were administered RHP and HHP at a dose of 126 mg kg^−1^, and rats in the CON group and MOD group were given the same volume of normal saline. Rats were orally administered continuously for 2 weeks. After the 12th day of oral administration, all the rats except for those in the CON group were injected with ISO (10 mg/kg, i.p.) for three consecutive days to cause AMI. On the 14th day, 1 h after the oral administration and the injection of ISO, the feces were collected. At the end of the experiment, the fecal samples from rats in all groups were quickly collected. Then rats were anesthetized with 1.2 g kg^−1^ ethyl carbamate. The blood and heart were collected. The blood samples were stood at room temperature for 0.5 ~ 1 h and then centrifuged at 4°C (1000×*g*, 15 min) to obtain the serum for analysis (3000 rpm, TGL‐16 equipped with 10 mL × 6 rotor, Sichuan Shuke Instrument Co. LTD).

### 2.4 Histological Analysis of Heart Tissue

After fixation in 4% paraformaldehyde, heart tissues were embedded in paraffin and sliced into 5 μm thick sections. These sections underwent hematoxylin–eosin (H&E) staining, and the resulting histopathological alterations in the heart were examined by an inverted microscope (Nikon Jiangnan Optical Instrument Co. Ltd., Nanjing, China).

### 2.5 Measurement of Oxidative Stress Markers and Myocardial Enzyme

The level of Malondialdehyde (MDA), activities of Superoxide dismutase (SOD) and Lactate dehydrogenase (LDH) were determined by biochemical kits. Serum CK, CK‐MB and cTn‐I concentrations were measured using ELISA kits. All of the experimental procedures were conducted according to the manuals' guidelines.

### 2.6 Fecal DNA Extraction and Gut Microbiota Analysis

Fecal DNA extraction was carried out using the TGuide S96. The structure of the intestinal microbiota was detected by high‐throughput 16S rDNA sequencing technology. Libraries were constructed and pooled according to the target downstream data volume requirement and effective concentration, followed by sequencing on the Beijing Baimaike platform.

The Raw data generated by Baimaike was preprocessed by Trimmomatic to produce clean data. These clean reads were then assembled using USEARCH. Further, gene prediction and abundance analysis were carried out utilizing QIIME software.

### 2.7 SCFAs Analysis

The concentration of acetic acid, propionic acid, and butyric acid was determined by HPLC method as previously described (Lou et al. 2024).

### 2.8 Statistical Analysis

The experimental data were expressed by mean ± standard deviation, and the data were processed statistically utilizing SPSS version 26.0. Independent‐sample *t*‐test was applied for pairwise comparisons, and one‐way analysis of variance (one‐way ANOVA) was applied for comparisons between multiple groups. LSD tests were applied for post hoc tests. For data with unequal variances, Tamhane's T2 tests were applied for post hoc tests. For data that did not follow a normal distribution, nonparametric tests were employed. Graph representations of the data were created from GraphPad Prism 8.0 software.

## 3 Result

### 3.1 Honey‐Processing Changed the Contents of OAC and TPC, as Well as the Character of Pectin in HH

The results indicated that OAC and TPC decreased, while TFC and RSC did not change after honey‐processing of hawthorn, as presented in Table 1.

**TABLE 1:** Contents of small molecular compounds in RH and HH ($\bar{X}$ ± SD, *n* = 3).

Sample	TFC/%	OAC/%	RSC/%	TPC/%
RH	7.22 ± 0.03	8.57 ± 0.04	23.86 ± 0.17	1.72 ± 0.04
HH	7.18 ± 0.01	6.02 ± 0.04**	25.05 ± 0.27	1.39 ± 0.02**

** *p* < 0.01 a significant difference between compounds in RH and HH.

Compared with RHP, the molecular weight (Mw) of HHP had no obvious change, but the DE, content of GalA, and the viscosity‐average molecular weight decreased significantly, as shown in Tables 2 and 3.

**TABLE 2:** Mw, DE, content of GalA and Mv of RHP and HHP($\bar{X\;}$± SD, *n* = 3).

Sample	Mw/kDa	DE/%	GalA/%	Mv/g/moL
RHP	11.97 ± 0.03	56.98 ± 0.03	86.03 ± 0.60	23,564 ± 104
HHP	11.39 ± 0.05	35.68 ± 0.72**	57.98 ± 0.30**	7290 ± 24**

*Note:* There is a significant difference between RHP and HHP (**p < 0.01 vs. RHP group).

### 3.2 RHP and HHP Administration Could Prevent Cardiac Damage Caused by ISO

**FIGURE 1:**
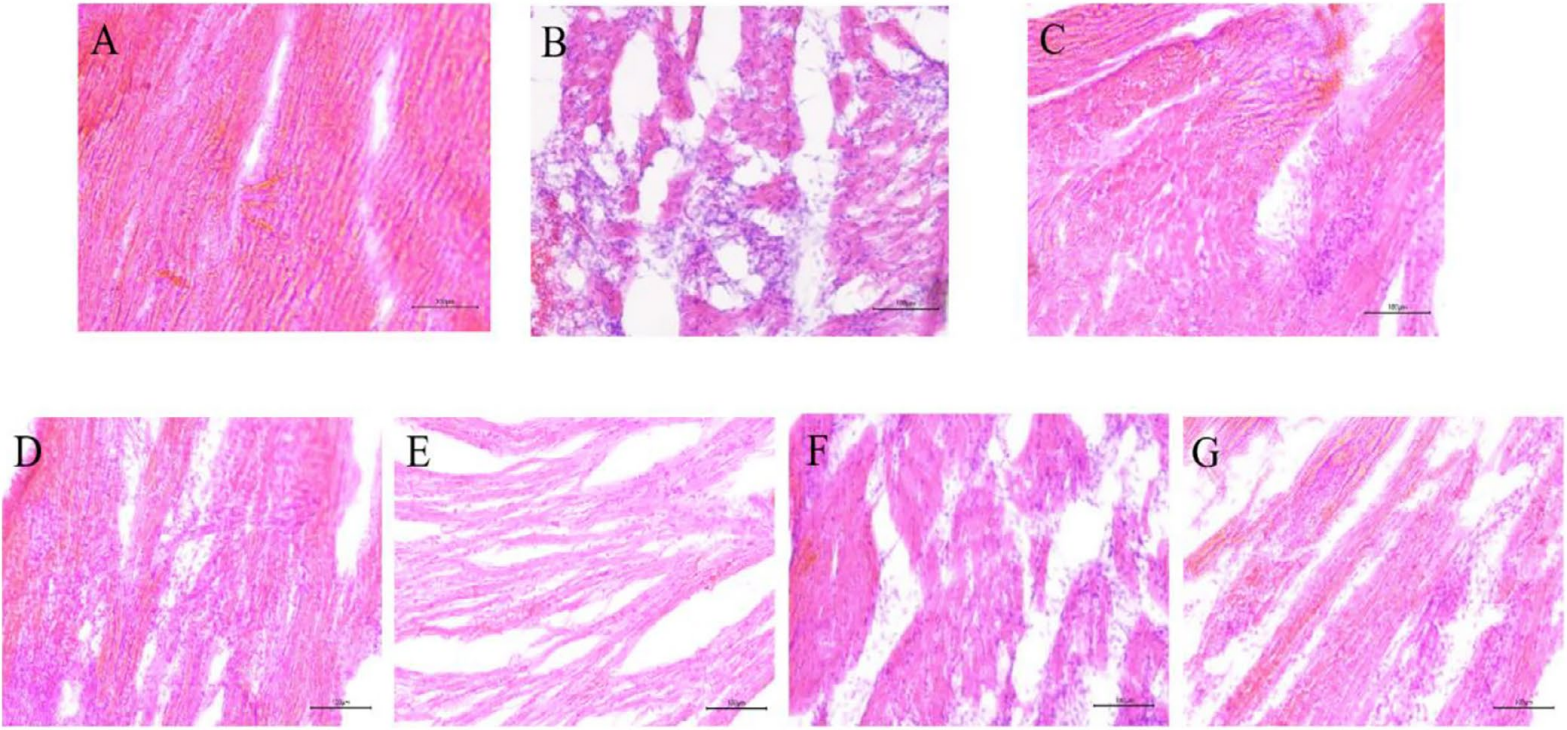
HE staining of rat heart tissue (A) CON group, (B) MOD group, (C) CDDP group, (D) RH group, (E) HH group, (F) RHP group, (G) HHP group (×200).

The cardiomyocytes in the CON group were neatly arranged, the texture was clear, the morphology and structure were normal, and there was no obvious injury and inflammation. While in the MOD group, the structure of cardiomyocytes was disordered and broken, the boundary was not clear, and the infiltration of inflammatory cells around blood vessels and myocardial interstitial swelled. These suggested that ISO induced cardiomyocyte damage. The cardiomyocytes in the RH and CDDP groups were neatly arranged as compared to those in the CON group. The cardiomyocytes in the HH and HHP groups were arranged neatly, but the structure was loose, and the therapeutic effect was weaker. The arrangement of cardiomyocytes in the RHP group was still disordered. See Figure 1 for details.

### 3.3 RHP and HHP Could Regulate Oxidative Stress Indicators and Myocardial Enzyme Indicators in AMI

**FIGURE 2:**
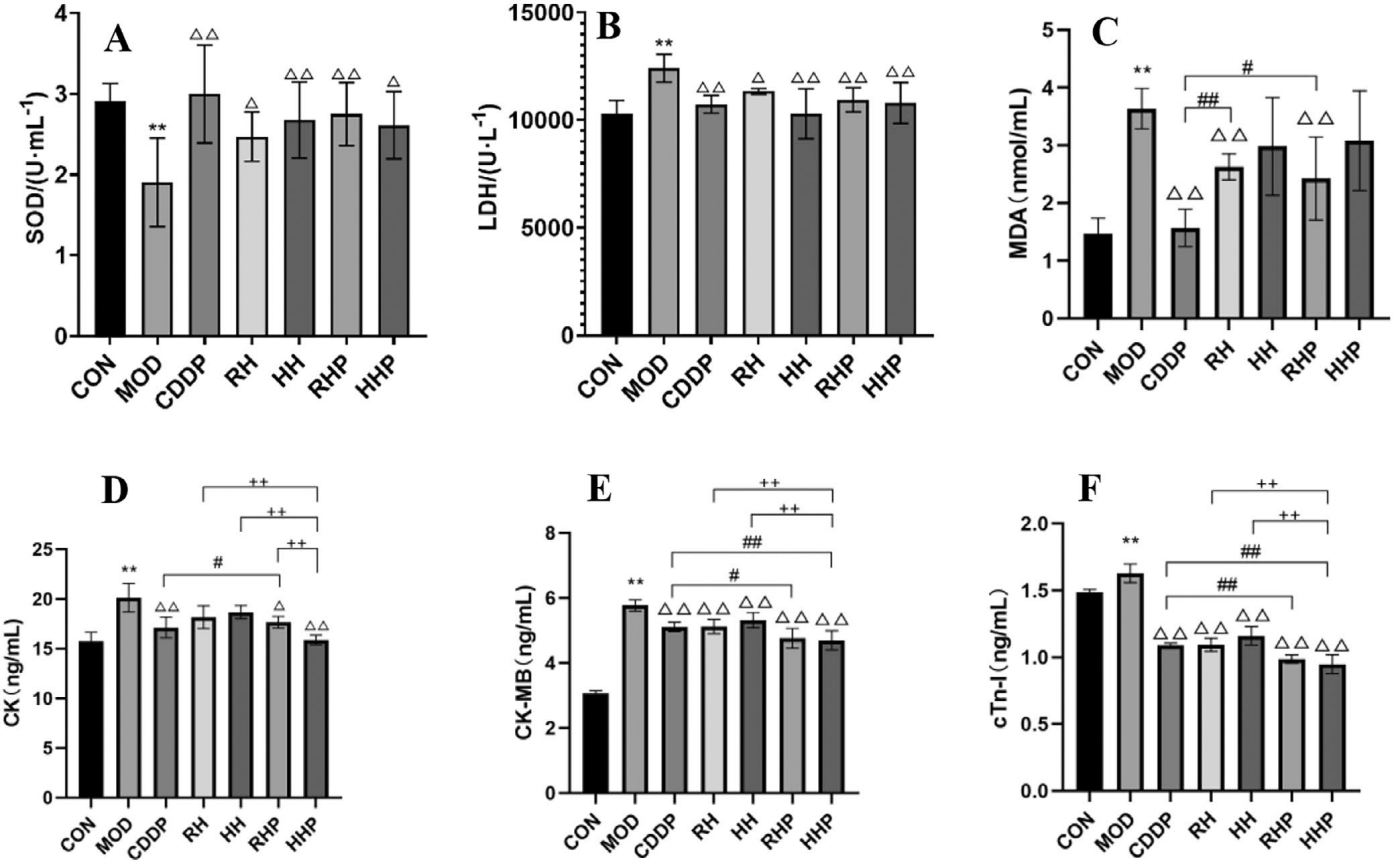
Biochemical indexes in rat serum. (A) SOD, (B) LDH, (C) MDA, (D) CK, (E) CK‐MB, (F) cTn‐I. *p < 0.05 and **p < 0.01 versus CON group, ^△^ *p* < 0.05 and ^△△^ *p* < 0.01 versus MOD group, #*p* < 0.05 and ##*p* < 0.01 versus CDDP group, +*p* < 0.05 and ++*p* < 0.01 versus each dosing group.

The results for each indicator showed that compared to the CON group, AMI injury was significantly manifested in the MOD group as reduced SOD activity and markedly increased levels of LDH, MDA, CK, CK‐MB, and cTn‐I. In the SOD, LDH, CK‐MB, and cTn‐I indicators, all drug administration groups showed significant regulatory effects compared to the MOD group. For SOD and LDH, the RH, HH, RHP, and HHP groups exhibited no significant difference in regulatory effects compared to the CDDP group. However, for CK‐MB and cTn‐I, the RHP and HHP groups demonstrated stronger regulatory effects than the CDDP group. Regarding CK, compared to the MOD group, the CDDP, RHP, and HHP groups showed significant regulatory effects, with the effect of the HHP group being similar to that of the CDDP group, while that of the RHP group was comparatively weaker. For MDA, compared to the MOD group, the CDDP, RH, and RHP groups displayed good therapeutic effects, though the regulatory effects of the RH and RHP groups were weaker than those of the CDDP group (Figure 2).

### 3.4 RHP and HHP Modulated Gut Microbiota Dysbiosis in AMI Rats

**FIGURE 3:**
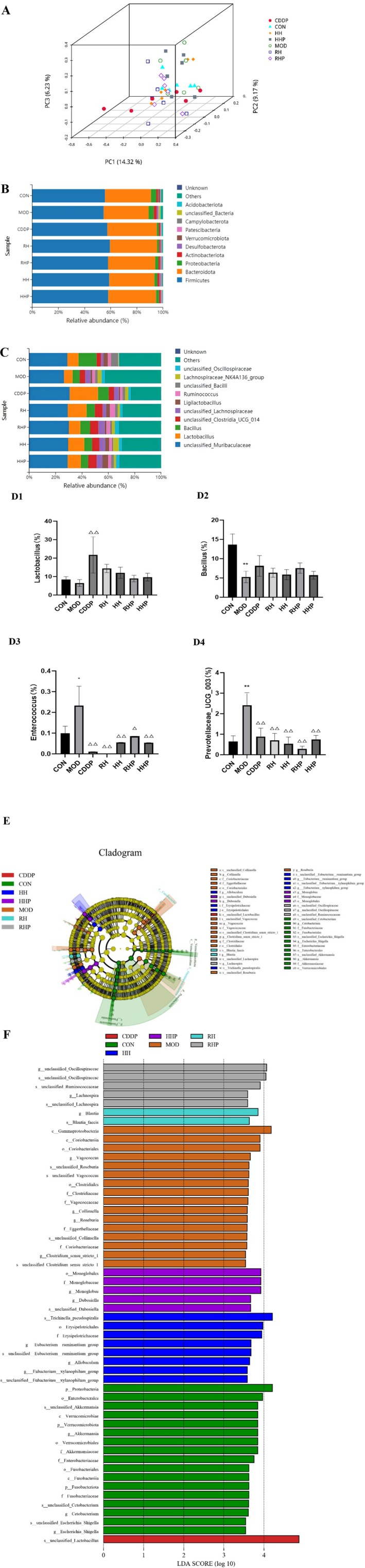
Diversity analysis of intestinal microflora in rats. (A) Principal co‐ordinates analysis (PCoA), stacked column of species abundance of intestinal microflora of rats at (B) phylum, (C) genus levels, (D1–4) bar plots of relative abundance of genus with significant difference between groups, (E) LEfSe analysis, and (F) linear discriminant analysis (LDA) of intestinal flora. *p < 0.05 and **p < 0.01 versus CON group, ^△^ *p* < 0.05 and ^△△^ *p* < < 0.01 versus MOD group.)

As shown in Figure 3, in the Principal Co‐ordinates Analysis (PCoA) diagram, the more similar the composition and structure of the flora among the samples, the closer the distance of the dots in the diagram. The result of PCoA showed that there were significant differences in the composition of intestinal flora between the groups.

*Firmicutes*, *Bacteroidota*, *Proteobacteria*, *Actinobacteria*, *Desulfobacterota*, *Verrucomicrobiota*, *Patescibacteria*, and *Campylobacterota* were identified from each group of samples. The relative abundance of *Firmicutes* was the highest, followed by *Bacteroidota*. There was no significant difference in the abundance of intestinal flora among the groups at the phylum level.

Several bacterial genera were identified from the groups, including *Lactobacillus*, *Bacillus*, *Enterococcus*, and *Prevotellaceae_UCG_003*. *Unclassified_Muribaculaceae* exhibited the relatively higher abundance, followed by *Lactobacillus*. In the MOD group, the relative abundances of *Bacillus* decreased compared to the CON group, while those of *Enterococcus* and *Prevotellaceae_UCG_003* increased. Conversely, in the CDDP group, the relative abundance of *Bacillus* and *Lactobacillus* increased compared to the MOD group. Whereas *Enterococcus* and *Prevotellaceae_UCG_003* decreased in all treatment groups. These results indicated that RH, HH, RHP, and HHP regulated the intestinal flora imbalance induced by AMI.

LEfSe analysis was used to detect the significant differences in the relative abundance of dominant microflora among different groups, and the differences of LDA score > 3.5 among seven groups were screened. At the genus level, *Akkermansia*, *Cetobacterium*, and *Escherichia_Shigella* were enriched in the CON group. *Vagococcus*, *Collinsella*, *Roseburia*, and *Clostridium_sensu_stricto_1* were enriched in the MOD group. *Blautia* was enriched in the RH group. *Eubacterium_ruminantium group*, *Allobaculum*, and *Eubacterium_xylanophilum group* were enriched in the HH group. *unclassified_Oscillospiraceae* and *Lachnospira* were enriched in the RHP group. *Monoglobus* and *Dubosiella* were enriched in the HHP group.

### 3.5 The Differences in SCFAs Regulated by RHP and HHP

Compared to the CON group, the MOD group showed a significant increase only in acetic acid, with no significant changes in propionic acid or butyric acid. When compared to the MOD group, all treatment groups exhibited significant alterations in acetic acid levels: CDDP, RH, RHP, and HHP groups significantly decreased acetic acid, with no notable difference in regulatory efficacy among them. In contrast, the HH group showed a significant increase in acetic acid, opposing the trend observed in other treatment groups. In the modulation of propionic acid, compared to the MOD group, both the HH and RHP groups showed significant changes in content but exhibited opposite trends: the HH group demonstrated an increase in propionic acid levels, whereas the RHP group displayed a decrease. No significant alterations in propionic acid were observed in the CDDP group or other treatment groups. Regarding the modulation of butyric acid, compared to the MOD group, the CDDP, HH, and HHP groups all showed significant increases in butyric acid content. Among these, the CDDP and HHP groups exhibited more pronounced regulatory effects, while the HH group demonstrated relatively weaker effects compared to these two groups (Table 3).

**TABLE 3:** Short‐chain fatty acid (SCFAs) contents in the feces (μg/g).

Groups	Acetic acid	Propionic acid	Butyric acid
CON	905.34 ± 84.17	311.32 ± 118.30	257.05 ± 45.47
MOD	1439.27 ± 41.70**	243.14 ± 15.34	292.92 ± 29.64
CDDP	1130.39 ± 85.06^△△△^	249.91 ± 36.86	546.15 ± 51.26^△△△^
RH	1248.93 ± 205.75^△^	204.91 ± 38.40	380.00 ± 82.92
HH	1684.12 ± 203.61^△△△^	471.85 ± 88.13^△△△^	476.07 ± 69.03^△^
RHP	1166.70 ± 77.51^△△△^	155.48 ± 59.57^△^	227.86 ± 38.23
HHP	1002.29 ± 155.61^△△△^	228.96 ± 59.34	553.42 ± 277.58^△△△^

*Note:* Data are presented as the mean ± SD (*n* = 6).

^△△△^ *P* < 0.01 versus MOD group.

^△^ *P* < 0.05 versus MOD group.

** *p* < 0.01 versus CON group.

## 4 Discussion

Hawthorn, a traditional Chinese medicine used as both a food and a therapeutic agent, contained flavonoids that exhibited antioxidant, antibacterial, and anti‐arteriosclerosis properties (Hu et al. 2023; Liu et al. 2025; Zhang et al. 2021). The total flavonoid content of hawthorn decreased with the extent of stir‐frying, ranking as follows: hawthorn > stir‐fried hawthorn > scorched hawthorn > charcoal from hawthorn (Chen 2023). After honey‐processing, the total flavonoid content of traditional Chinese medicine usually decreased (He et al. 2023; Song et al. 2013). The literature attributed this to the increased viscosity of decoction pieces and thus reduced the dissolution of active components (He et al. 2023). But several studies have demonstrated that honey‐processing increased the content of total flavonoid in traditional Chinese medicine. The addition of honey appeared to inhibit the transformation of chemical components induced by thermal processing, thereby stabilizing the structure of the compounds in traditional Chinese medicine (Chen et al. 2018). Conversely, flavonoid monomer compounds could degrade from polysaccharides glycosides to their monoglycosides and aglycones during heating. Additionally, glycosides might form through the combination of aglycones and saccharides during honey‐processing. Therefore, the content change of flavonoids in HH might be influenced by the specific structures involved (Ao et al. 2020; Yang and Yang 2012).

The content of organic acids in hawthorn was second only to flavonoids. These compounds were known to help dilate blood vessels and reduce blood pressure (Yuan et al. 2024). Compared to hawthorn, the total organic acid content in HH was lower, likely due to the decomposition and transformation of organic acids during honey‐processing (Chen and D 2016). RH contains fructose, glucose, and other reducing sugars (Liu et al. 2010), while honey also contains similar sugars (Li et al. 2023). Consequently, under the experimental conditions, the content of reducing sugars in the HH increased slightly with an insignificant difference. Pectin was a macromolecular soluble heteropolysaccharide commonly found in plant cells. Its main components included homogalacturonan (HG), rhamnogalacturonic acid II (RG‐II), and rhamnogalacturonic acid I (RG‐I), which exhibited various biological activities and were widely utilized in the biological, pharmaceutical, and food industries (Rahmani et al. 2020). The decrease in the esterification degree of HHP may be attributed to the saponification of the methoxy groups during the heating extraction process (Houben et al. 2011). The depolymerization of the HG region in pectin during the heating process converted water‐insoluble pectin polysaccharides in the cell wall into water‐soluble pectin, thereby reducing the content of GalA in pectin (Zhou et al. 2021).

The symptoms of AMI are comparable to those of chest pain, heartache, and syncope as described in traditional Chinese disease (Kaski et al. 2018). Oxidative stress and the inflammatory response were two critical factors in the pathophysiology of myocardial ischemia (Yang et al. 2019). During myocardial ischemia, the myocardium rapidly shifted from aerobic to anaerobic metabolism, leading to pathological changes, including arrhythmias, abnormal myocardial cell function, and energy metabolism disorders (Boccaletto et al. 2022; Motorin and Helm 2022). MDA was a biomarker associated with elevated levels of free radicals, reflecting early abnormal cellular metabolism and disease progression (Sri Iswari et al. 2021). SOD was commonly used as a marker for cellular oxidative damage. It catalyzed the disproportionation of superoxide anions to produce hydrogen peroxide (H_2_O_2_) and molecular oxygen (O_2_), which played crucial roles in scavenging free radicals in biological systems (Ren et al. 2019). CK, LDH, and CK‐MB were established biomarkers of myocardial injury. These markers significantly increase during myocardial damage (Li 2024). RHP and HHP offered superior protection against myocardial injury induced by AMI compared to RH and HH, as evidenced by their ability to regulate the levels of CK, CK‐MB, and cTn‐I. Additionally, both RH and RHP demonstrated greater efficacy than HH and HHP in inhibiting the production of MDA. The primary antioxidant component in RH was phenolic compounds (Wen et al. 2015). After being honey‐processed, the TPC in hawthorn is lower compared to that in unprocessed hawthorn; this might explain why RH is superior to HH in this regard. Additionally, the GalA in pectin shows a positive correlation with its antioxidant activity (Ogutu and Mu 2017). Consequently, RHP was superior to HHP in the antioxidant index MDA. A similar result was found in our previous research, that regulation on MDA of FHP on AMI rats was weaker than RHP (Lou et al. 2024). Unlike our previous results that FHP, low DE pectin, showed a better regulation effect on CK, CK‐MB, and cTn‐I, HHP, also as low DE pectin, herein showed similar effects on CK‐MB and cTn‐I as compared with those of RHP, and only a better regulation on CK was observed. So the structure and cardioprotective effect relationship of hawthorn pectin needs further elucidation.

SCFAs were the primary metabolites of dietary fiber and carbohydrates resulting from anaerobic fermentation in the colon. Acetic acid, propionic acid, and butyric acid collectively comprised approximately 95% of SCFAs, with their typical proportion being 3:1:1 (Lv et al. 2022). In this experiment, the ratio of acetic acid, propionic acid, and butyric acid in the feces of rats in the CON group was approximately 3:1:1, indicating a balanced intestinal microbiota in this group.

The composition of intestinal microbiota was similar in humans and rats, primarily composed of *Bacteroidota*, *Firmicutes*, *Proteobacteria*, and *Actinobacteria*. Among these, *Firmicutes* and *Bacteroidota* were considered beneficial bacteria, collectively accounting for over 90% of the intestinal microbiota (Tang et al. 2017). In response to acute disease, such as myocardial ischemia–reperfusion (MIR), damage to the intestinal barrier altered the microbiota composition, leading to an increase in *Proteobacteria* (Zhao et al. 2023). Additionally, AMI induced by arterial ligation led to the change in the diversity and abundance of gut microbiota, i.e., alpha‐diversity (Wu et al. 2017). Furthermore, AMI induced by ISO (i.p. 85 mg/kg) over two continuous days led to the decrease of Firmicutes and the increase of Bacteroidetes and Spirochaetae (Sun et al. 2019). In the present research, AMI was induced by ISO (i.p. 10 mg/kg). The different method of modeling led to the insignificant difference in the abundance of intestinal flora among the groups at the phylum level (Lou et al. 2024). However, the beta‐diversity observed from PCoA indicated the different composition in intestinal microbiota among CON, MOD, and drug administration groups.

According to the results of LEfse, *Vagococcus*, *Clostridium_sensu_stricto_1*, *Collinsella*, and *Roseburia* were enriched in the MOD group. As a type of Gram‐positive bacterium, *Vagococcus* has been found to infect various animals, leading to fibrin deposition in organs such as the spleen and liver, as well as visceral bleeding and septicemia (Zheng et al. 2017). *Clostridium_sensu_stricto_1* posed a threat to humans, animals, and plants. Additionally, *Collinsella* destroyed the intestinal barrier and was associated with increased intestinal permeability (Wei et al. 2023). This observation is consistent with the literature suggesting that AMI could result in alterations in intestinal flora and subsequent mucosal injury (Zhao et al. 2023). Furthermore, a significant increase in the abundance of *Enterococcus* was observed in the MOD group. *Enterococci* were a facultative anaerobe with homofermentative metabolism, with lactic acid as the predominant end product of carbohydrate fermentation (García‐Solache and Rice 2019). The enrichment of facultative anaerobes *Vagococcus* and *Enterococcus* indicated the change of the metabolic pattern of colonocyte under AMI (Litvak et al. 2018). The relative abundance of *Bacillus* in the MOD group decreased, while the relative abundance of *Prevotellaceae_UCG_003* increased. *Bacillus subtilis* , a member of the *Bacillus* genus, was recognized for its probiotic properties in poultry farming, where it enhanced the production of acetic and butyric acids in the ceca of broilers (Xu et al. 2021). *Prevotella* was a beneficial bacterium associated with high‐fiber diets, capable of utilizing polysaccharides to promote the synthesis of SCFAs, particularly acetic acid and succinic acid (Ma et al. 2025). Previous studies indicated the increase of *Prevotellaceae_UCG_003* might relate to the severity of inflammation in ISO‐induced AMI (Sun et al. 2019). Additionally, the MOD group showed enrichment of *Roseburia*, a Gram‐positive anaerobe known for its high butyric acid production, which contributed to the inhibition of intestinal inflammation through the generation of beneficial metabolites (Kang et al. 2023). Under ischemic or hypoxic conditions, glycolysis became a critical pathway for ATP production. The increased level of acetic acid in the MOD group was attributed to the increased abundance of SCFAs‐producing bacteria and thus to improve the intestinal imbalance as a spontaneous response of the organism to AMI. Notably, the populations of *Prevotellaceae_UCG_003* and *Enterococcus* significantly declined across all treatment groups, indicating their potential role in mitigating intestinal flora imbalances associated with AMI.

*Blautia*, enriched in the RH group, was an anaerobic bacterium with probiotic properties. It was essential for maintaining intestinal environmental balance and preventing inflammation by upregulating intestinal regulatory T cells and producing SCFAs, including acetic acid, succinic acid, and lactic acid (Liu et al. 2021). *Allobaculum*, enriched in the HH group, showed a positive correlation with the levels of propionic acid and butyric acid in the cecum of insulin‐resistant diabetic mice (Pujo et al. 2021; Vallianou et al. 2021). Furthermore, the HH group enriched two *Eubacterium* species: *Eubacterium_ruminantium group* and *Eubacterium_xylanophilum group*, both of which could produce butyric acid (Kim et al. 2020; Soto‐Martin et al. 2020; Zagato et al. 2020). Notably, the proportion of the three types of SCFAs in the feces of the HH group was similar to that of the CON group, specifically in a ratio of 3:1:1. This suggested that the HH group played a more effective role in maintaining intestinal homeostasis through the regulation of intestinal flora. *Lachnospira*, an anaerobic pectinolytic bacterium, was enriched in the RHP group and was capable of fermenting dietary polysaccharides to yield SCFAs in the gastrointestinal tract. *Monoglobus*, which was prevalent in the HHP group, served as a specialized pectin‐degrading and butyrate‐producing bacterium in the colon (Chen et al. 2022; Kim et al. 2019). The proportion of acetic acid in the RHP group was significantly higher than that in the CON group, while the HHP group exhibited a significantly higher proportion of butyric acid compared to the CON group. These differences might be attributed to variations in GalA and DE between the two groups, leading to the enrichment of distinct bacterial populations and the production of diverse metabolites (Larsen et al. 2019). Notably, the HH and the HHP groups enriched *Allobaculum* and *Monoglobus*, respectively, which might explain the significant increase in butyric acid levels observed in the feces of these groups. As a primary energy source for colonic epithelial cells, butyric acid played a crucial role in mitigating the inflammatory responses and oxidative stress associated with myocardial ischemia induced by isoproterenol (Lu et al. 2024).

The findings of this study indicated that RH, HH, and their pectin could mitigate myocardial injury in rats with ISO‐induced AMI. Specifically, RHP and HHP exhibited greater efficacy than RH and HH in down‐regulating CK and CK‐MB levels. Additionally, RH and RHP outperformed HH and HHP in regulating MDA levels. Each treatment group significantly altered the diversity of intestinal microbiota and the contents of SCFAs in the feces of rats suffering from AMI. Moreover, HH demonstrated superior effectiveness compared to RH in maintaining intestinal microflora homeostasis and SCFAs proportions. Notably, RHP and HHP enriched the genera *Lachnospira* and *Monoglobus*, respectively, leading to differing SCFAs proportions of these two treatment groups.

To summarize, the gut‐microbiota‐heart axis was pivotal for the development of AMI (Panyod et al. 2021). By affecting the structure, abundance, and metabolites of the gut flora, HHP promoted the content of gut flora‐derived SCFAs, which were key molecules of the gut‐microbiota‐brain axis. While our study proposed a possible mechanism through which HHP exerted its anti‐AMI effects, it has not been fully validated. This limitation highlighted the need for further detailed investigations, microbiota transplantation experiments for example, to confirm whether these pathways represented the main mechanisms of action for HHP and to elucidate the primary role of the gut microbiota‐heart axis.

## Author Contributions

**Jing Li:** writing – original draft (lead). **Meiqi Liu:** data curation (equal). **Yu Zheng:** writing – review and editing (equal). **Qian Cai:** writing – review and editing (equal). **Baojie Zhang:** investigation (equal). **Yang Qu:** writing – review and editing (equal).

## Ethics Statement

Ethical approval to report this case was obtained from the Ethics Committee of the National Research Council's Guidelines for the Care and Use of Experimental Animals and the Research Ethics Committee of Liaoning University of Traditional Chinese Medicine, license number 210000420220209, 8\25\2022.

## Conflicts of Interest

The authors declare no conflicts of interest.

## Data Availability

Data available on request from the authors.
